# Dietary Nutritional Information Autonomous Perception Method Based on Machine Vision in Smart Homes

**DOI:** 10.3390/e24070868

**Published:** 2022-06-24

**Authors:** Hongyang Li, Guanci Yang

**Affiliations:** 1 Key Laboratory of Advanced Manufacturing Technology of the Ministry of Education, Guizhou University, Guiyang 550025, China; lihongyang159951@163.com; 2 Key Laboratory of “Internet+” Collaborative Intelligent Manufacturing in Guizhou Province, Guiyang 550025, China; 3 State Key Laboratory of Public Big Data, Guizhou University, Guiyang 550025, China

**Keywords:** nutritional information, autonomous perception, YOLOv5, social robot, smart home

## Abstract

In order to automatically perceive the user’s dietary nutritional information in the smart home environment, this paper proposes a dietary nutritional information autonomous perception method based on machine vision in smart homes. Firstly, we proposed a food-recognition algorithm based on YOLOv5 to monitor the user’s dietary intake using the social robot. Secondly, in order to obtain the nutritional composition of the user’s dietary intake, we calibrated the weight of food ingredients and designed the method for the calculation of food nutritional composition; then, we proposed a dietary nutritional information autonomous perception method based on machine vision (DNPM) that supports the quantitative analysis of nutritional composition. Finally, the proposed algorithm was tested on the self-expanded dataset CFNet-34 based on the Chinese food dataset ChineseFoodNet. The test results show that the average recognition accuracy of the food-recognition algorithm based on YOLOv5 is 89.7%, showing good accuracy and robustness. According to the performance test results of the dietary nutritional information autonomous perception system in smart homes, the average nutritional composition perception accuracy of the system was 90.1%, the response time was less than 6 ms, and the speed was higher than 18 fps, showing excellent robustness and nutritional composition perception performance.

## 1. Introduction

Along with the gradual development of IoT, big data and artificial intelligence, smart homes are changing people’s lives and habits to a certain extent [1]. According to data released by Strategy Analytics, since 2016, the number of households with smart home devices in the world and the market size of smart home devices have both continued to grow. In 2020, the global smart home equipment market will reach 121 billion US dollars, and the number of households with smart home equipment in the world will reach 235 million. In addition, deep learning has brought state-of-the-art performance to tasks in various fields, including speech recognition and natural language understanding [2], image recognition and classification [3], system identification and parameter estimation [4,5,6].

According to the 2020 World Health Organization (WHO) survey report, obesity and overweight are currently critical factors endangering health [7]. Indisputably, obesity may cause heart disease, stroke, diabetes, high blood pressure and other diseases [8]. Since 2016, more than 1.9 billion adults worldwide have been identified as overweight, especially in the United States. In 2019, the rate of obesity in all states was more than 30%, and such patients spent USD 1429 more a year on medical diseases than normal people [9]. Six of the ten leading causes of death in the United States, including cancer, diabetes and heart disease, can be directly linked to diet [10]. Though there are various factors that may cause obesity such as certain medications, emotional issues such as stress, less exercise, poor sleep quality, and eating behavior—what and how people eat is always the major problem that results in weight gain [11].

Likewise, among China’s residents, there are health problems such as obesity, unbalanced diets and being overweight [12]. According to the 2020 *Report on Nutrition and Chronic Diseases of Chinese Residents*, the problem of being overweight and obese among residents has become increasingly prominent, and the prevalence and incidence of chronic diseases are still on the rise [13]. Correspondingly, the Chinese government provides dietary guidance and advice to different groups of people by issuing dietary guidelines to help them eat properly, thereby reducing the risk of disease [14]. There is no doubt that dietary behavior is a key cause of obesity, and nutritional composition intake is an important index to measure whether the diet is excessive or healthy. Methods such as “24-h Diet recall” are traditional methods of diet quality assessment [11], but it is difficult to guarantee their accuracy because of the subjective judgment and estimation deviation of users [15]. Thus, a variety of objective visual-based dietary assessment approaches, ranging from the stereo-based approach [16], model-based approach [17,18], depth-camera-based approach [19] and deep learning approaches [20], have been proposed. Despite these methods having shown promises in food volume estimation, several key challenges, such as view occlusion and scale ambiguity, are still unresolved [21]. In addition, over-reliance on personal tastes and preferences will lead to nutritional excess or nutritional imbalance, which can lead to various chronic diseases [22].

In order to automatically perceive the user’s dietary information in the smart home environment, this paper proposes a dietary nutritional information autonomous perception method based on machine vision in smart homes (DNPM). We only need to recognize the user’s diet through the camera, then associate its nutritional composition, measure the user’s daily nutritional composition, and ensure the user’s healthy diet for a long time. The main contributions are summarized as follows.

- In order to monitor the user’s dietary intake using the social robot, we proposed a food-recognition algorithm based on YOLOv5. The algorithm can recognize multiple foods in multiple dishes in a dining-table scenario and has powerful target detection capabilities and real-time performance.
- In order to obtain the nutritional composition of the user’s diet, we calibrated the weight of food ingredients and designed the method for the calculation of food nutritional composition; then, we proposed a dietary nutritional information autonomous perception method based on machine vision (DNPM) that supports the quantitative analysis of nutritional composition.
- Deployed the proposed algorithms on the experimental platform and integrate it into the application system for testing. The test results show that the system shows excellent robustness, generalization ability and nutritional composition perception performance.

The remainder is arranged as follows. Section 2 introduces the related work, Section 3 introduces the food-recognition algorithm, Section 4 focuses on the proposed method, and Section 5 details the experimental environment. Section 6 presents the performance experiment and analysis of the food-recognition algorithm. Section 7 discusses the performance testing and analysis of the application system. Section 8 is a necessary discussion of the results of this paper. Finally, in Section 9 we conclude this paper and discuss possible future works.

## 2. Related Work

In the last decade, the perception technology of dietary nutritional composition has been widely researched by scholars at home and abroad. Researchers have carried out a series of studies on food dataset construction, food recognition and diet quality assessment.

In the construction of food datasets, training data are mainly collected by manual annotation methods to construct a food image dataset in the early stages [23,24,25]. However, the method based on the manual labeling of datasets is expensive and poorly scalable. Moreover, coupled with factors such as variable image shooting distances and angles, and mutual occlusion among food components, it is difficult to guarantee the accuracy of artificial image classification standards. Compared with the data obtained based on manual annotation methods, Bossard L. et al. [26] collected 101,000 food images containing 101 categories from food photo-sharing websites and established the ETH Food-101 dataset; however, one image often inevitably contains multiple foods. ChineseFoodNet [27] consists of 185,628 Chinese food images in 208 categories, but no fruit images are involved, and the definition of image categories in the dataset is relatively vague. Parneet et al. [28] constructed the FoodX-251 dataset based on the Food-101 public dataset for fine-grained food classification, which contains 158,000 images and 251 fine-grained food categories, although most of them are Western-style food.

In terms of food recognition, Matsuda et al. [29] incorporated the information on co-occurrence relationships between foods. Specifically, four kinds of detectors are used to detect the candidate regions of the image; then, the candidate regions are fused. After extracting a variety of image features, the images are classified, and the manifold sorting method is adopted to identify a variety of foods. Zhu et al. [30] developed a mobile food image recognition method. Firstly, the food region in the image is located by the image segmentation method; then, the color and texture features of the region are extracted and fused for food image recognition. Kong et al. [31] provided a food recognition system DietCam, which extracts SIFT features as food image features with characteristics such as illumination, scale and affine invariance, and obtains three food images from three different shooting angles at a time; then, it performs more robust recognition based on these three food images. However, the existing food image recognition methods are mainly aimed at a single task, such as food classification, while there are few studies on simultaneously predicting food ingredients’ energy and other information corresponding to food images. Food image recognition can be improved by learning food categories and food ingredients’ attributes at the same time through multi-task learning. Dehais et al. [32] performed a 3D reconstruction of food based on multi-angle images to predict the carbohydrate content of food. However, the food volume estimation method based on multi-angle images requires the input of multiple images and has higher requirements for shooting distance and angle, which is not convenient for users to operate. Myers and Johnston et al. [33] designed a mobile app called Im2Calories that predicts calorie values based on food images. Firstly, the food category is recognized by the GoogLeNet model; then, the different foods in the image are identified and located by target recognition, semantic segmentation, and the food volume is estimated based on the depth image. Finally, the calorie value is calculated by querying the USDA food information database. However, the related information has to be ignored in the training process, because the sub-tasks are independent of each other.

In fact, the quality assessment of a user’s diet can be further completed according to the associated components of the food image. Regarding diet quality assessment, Javaid Nabi et al. [34] proposed a Smart Dietary Monitoring System (SDMS) that integrates Wireless Sensor Networks (WSN) into the Internet of Things, tracks user’s dietary intake through sensors and analyzes data through statistical methods, so as to track and guide user’s nutritional needs. Rodrigo Zenun Franco [35] designed a recommendation system for assessing dietary intake, which systematically integrates the individual user’s dietary preferences, population data and expert recommendations for personalized dietary recommendations. Abul Doulah et al. [36] proposed a sensor-based dietary assessment and behavioral monitoring method in 2018 that obtains the user’s dietary intake through video and sensors, as well as differentiated statistics on eating time, intake time, intake times and pause time between eating times for the assessment of the user’s diet. In 2020, Landu Jiang et al. [11] developed a food image analysis and dietary assessment system based on the depth model, which was used to study and analyze food projects based on daily dietary images. In general, the dietary monitoring and assessment systems proposed above can track and monitor the user’s dietary behavior and assess the dietary intake, but cannot effectively assess the user’s diet quality. More fundamentally, the proposed systems do not correlate food image recognition algorithms, nor do they fully consider the main components of the diet; moreover, the food analyzed is too simple.

However, it is worth explaining that only by building an expanded multi-food dataset, realizing the multi-target recognition of foods to deal with complex life scenarios and making qualitative and quantitative analyses of the intake can we accurately assess the dietary intake of user’s and guide them toward healthier lifestyle choices. Though dataset construction, food recognition and diet quality assessment have been well discussed in the above work, three fundamental challenges remain. Firstly, most dataset images have only one type of food, and most methods of food recognition deal with images of a single food. Secondly, it is still time-consuming (2 s in general) to detect and classify the food in images. Finally, there is a lack of effective assessment of the user’s diet quality. In this paper, we aim to address these issues and propose a dietary nutritional information autonomous perception method based on machine vision (DNPM), recognizing foods through cameras, and correlating food nutritional composition to generate diet quality assessments for long-term healthcare plans.

## 3. Food-Recognition Algorithm Based on YOLOv5

**Figure 1:**
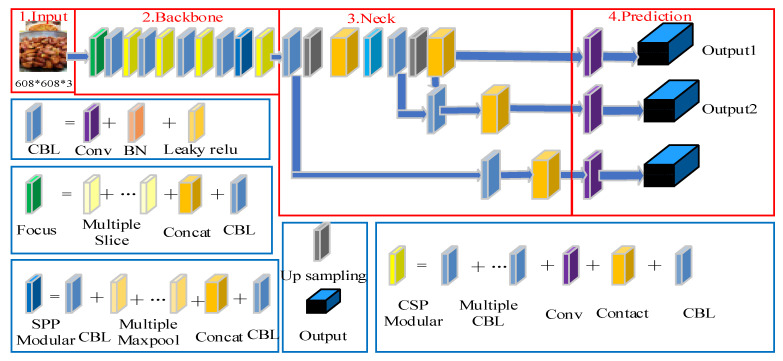
Overall architecture diagram of food-recognition algorithm based on YOLOv5.

In order to recognize multiple foods in multiple dishes in a dining-table scenario, using the powerful multi-target detection capability of YOLOv5 [37], we propose a food-recognition algorithm based on YOLOv5. Its overall architecture diagram is shown in Figure 1, and its detailed steps are shown in Algorithm 1.

In Figure 1, the Input layer preprocesses the training dataset through the Mosaic data-enhancement method, adaptive anchor-frame calculation, adaptive picture scaling and other methods [38]; it initializes the model parameters and obtains the required picture size of the model. The Backbone layer divides the picture in the dataset through the Focus structure; then, it scales the length and width of the image continuously through the CSP structure. The Neck layer fuses the data set through FPN operation and PAN operation to obtain the prediction feature map of the dataset. The Precision layer calculates the gap between the prediction box and the real box through the calculation of the loss function; then, it updates the parameters of the iterative model through the back-propagation algorithm and filters the prediction box through the NMS operation weighted by the model post-processing operation to obtain the prediction results of the model.
**Algorithm 1.** Food-recognition algorithm based on YOLOv5.
**Input:** training dataset *f* = {*f*_1_, *f*_2_, …, *f_n_*}, where *n* represents the total number of samples in the dataset;
**Output:** food-recognition database *F* = {*F*_1_, …, *F_i_*, …, *F_n_*};1:Initialize batch size = 32 and learning rate = 0.001;2:Input the food training dataset *f* = {*f*_1_, *f*_2_, …, *f_n_*} into the Input layer, and use Mosaic to perform data-enhancement operations such as random cutting and random distribution to obtain the dataset *C* = {*c*_1_, *c*_2_, …, *c_n_*};3:Use adaptive anchor box calculation to perform initial anchor box calibration on the training dataset *C* = {*c*_1_, *c*_2_, …, *c_n_*};4:Use adaptive image scaling technology to uniformly modify the size of the image to 608 × 608 × 3, and obtain the dataset *D* = {*d*_1_, *d*_2_, *d*_3_, …, *d_n_*};5:Input *D* = {*d*_1_, *d*_2_, *d*_3_, …, *d_n_*} into the Backbone layer. Use the Focus structure for segmentation and splicing to generate a 304 × 304 × 12 feature map. Then, through the CBL convolution unit, convolution is used for feature extraction. At the same time, the weight is normalized to reduce the amount of calculation, and finally becomes a 304 × 304 feature map vector *I* = {*I*_1_, *I*_2_, …, *I_n_*} through the Leaky Relu layer;6:process the 304 × 304 feature map vector *I* = {*I*_1_, *I*_2_, …, *I_n_*} through multiple CBL convolution units. Then, input to the CSP unit for feature extraction again. Inside, the feature map vector is divided into two parts. First, the feature graph is merged by Contact operation, BN operation, Relu activation function and CBL convolution operation, and then the downsampling operation is carried out by CBL convolution operation, multiple maximum pool operation, Contact operation, CBL convolution operation and so on, and the 19 × 19 feature map vector *M* = {*M*_1_, *M*_2_, …, *M_n_*} is obtained;7:Input *M* = {*M*_1_, *M*_2_, …, *M_n_*} into the Neck layer, and perform feature extraction again through CSP module, CBL convolution operation and upsampling to obtain feature vector *N* = {*N*_1_, *N*_2_, …, *N_n_*};8:Input *N* = {*N*_1_, *N*_2_, …, *N_n_*} into the FPN structure for feature splicing. The FPN structure includes Contact operation, CBL convolution operation, CSP module operation, etc. By means of the FPN structure transferring strong semantic features from top to bottom, and the PAN structure transferring strong localization features from bottom to top, the feature fusion is performed on the high-level feature information of the image, and the feature map vector *G* = {*g*_1_, *g*_2_, …, *g_n_*} of 19 × 19, 38 × 38, 76 × 76 is obtained;9:Input the feature map *G* = {*g*_1_, *g*_2_, …, *g_n_*} to the Prediction layer. The Prediction layer calculates the difference between the prediction frame and the real frame by calculating the loss, mainly the classification loss $y^{i}=Sigmoid\left(x^{i}\right)=\frac{1}{1+e^{-x_{i}}},\;L_{class}=-\overset{N}{\underset{n=1}{\sum }}y_{i}^{*}\log\left(y_{i}\right)+\left(1-y_{i}^{*}\right)\log\left(1-y_{i}\right)$ and regression loss $L_{GIOU}\left(B,B_{gt}\right)=1-\frac{B\cap B_{gt}}{B\cup B_{gt}}+\frac{\left|C-\left(B\cup B_{gt}\right)\right|}{\left|C\right|}$, and then reversely updates the iterative model parameters;10:The model algorithm will generate multiple prediction boxes, use the weighted NMS operation to filter the prediction boxes, and finally get the model prediction result dataset *F* = {*F*_1_, …, *F_i_*, …, *F_n_*}.

## 4. Dietary Nutritional Information Autonomous Perception Method Based on Machine Vision (DNPM)

In order to obtain the nutritional composition of foods, the weight of food ingredients needs to be calibrated first, and the standard weight of each food ingredient is calibrated according to the amount of food ingredients of “Meishi Jie” [39] (see Table 1). The nutritional composition of each food with a weight of 100 g is queried according to the National Nutrition Database-Food Nutritional Composition Query Platform [40] and Shi An Tong-Food Nutritional Composition Query Platform [41], and the recognized food is mapped to the nutritional composition table.

Assuming that there are *c* kinds of main ingredients to form a dish, and the standard nutritional composition of the *j*th ingredient is *Y_ij_*, then the nutritional composition of the *j*th ingredient *N_ij_* = *Y_ij_* × *G_j_*/100, where *G* represents the calibrated weight of ingredients, *i* = 1, 2, 3, 4, 5, …, 33 represent 33 nutritional compositions (see Table 2), *j* = 1, 2, …, *c* represent the *c* main ingredients of the dish (see Table 1).

The nutritional compositions of the main ingredients in the dish are accumulated to obtain the nutritional composition of the dish. The calculation method is shown in Equation (1):

(1) $$CP_{i}=\sum N_{ij}$$

where *CP_i_* represents the *i*th nutritional composition of the dish, *i* = 1, 2, …, 33.

Using Algorithm 1, the robot can obtain the feature model *w* of food recognition, that is, the food-recognition database *F* = {*F*_1_, …, *F_i_*, …, *F_n_*}. In order to obtain the food nutritional composition consumed by each user after the robot recognizes foods and faces through vision, we propose a dietary nutritional information autonomous perception method based on machine vision (DNPM), where the specific steps are shown in Algorithm 2.
**Algorithm 2.** Dietary nutritional information autonomous perception method based on machine vision.
**Input**: camera video stream *C* = {*c*_1_, *c*_2_, …, *c_n_*};Face data feature database *P* = {*p*_1_, *p*_2_, …, *p_k_*};Food feature model *w*;Food set *f* = {*f*_1_, *f*_2_, …, *f_z_*}, where the nutritional composition set of the *i*th food is *d_i_*;Nutritional composition database *D* = {*d*_1_, *d*_2_, *d_i_*, …, *d_z_*};The taboo food database *G* = {*g*_1_, *g*_2_, …, *g_k_*}, the taboo food of the *i*th person is *g_i_*;**Output**: Time *T* of this meal, food intake database *F_T_* = {*F*_1_, …, *F_i_*, …, *F_k_*}, nutritional composition intake database *D_T_* = {*D*_1_, …, *D_i_*, …, *D_k_*};1:Load face data feature database *P*, food feature model *w*, food set *f*, and nutritional composition database *D*; and initialize *t*_b_ = current time;2:Capture the frame data *I* = {*I*_1_, *I*_2_, …, *I_n_*} at the same moment in the video stream of camera *C* = {*c*_1_, *c*_2_, …, *c_n_*}, and set the temporary food set *T*_food_ = ∅, the temporary personnel set *T_p_* = ∅;3:Based on the food feature model *w*, use YOLOv5 to recognize the food items in *I* = {*I*_1_, *I*_2_, …, *I_n_*} to obtain the food set *T*_food_;4:Based on the face data feature database *P*, use the face recognition method to recognize the personal information in *I* = {*I*_1_, *I*_2_, …, *I_n_*}, and obtain the temporary personnel set *T_p_*;5:If *T*_food_ ! = ∅ && *T_p_* ! = ∅, turn to Step 6;  Else, turn to Step 2;6:For each person *p_i_* in *T_p_*, // Get the identity of the person at this table;   *D_i_* = ∅;   *F_i_* = ∅;For each food *f_i_* in *T*_food_, // Get the nutritional compositions of this table food;     if *f_i_* ∉ g*_i_*, *D_i_* = *D_i_*∪*d_i_* and *F_i_* = *F_i_*∪*f_i_*;      *D_T_* = *D_T_*∪*D_i_*;      *F_T_* = *F_T_*∪*F_i_*;7:Output *T = t_b_*, *D_T_* and *F_T_*.

In Step 4, firstly, capture face information and person name information in advance using the camera and store them locally; then, extract 128D feature values from multiple face images using the face database Dlib; calculate the 128D feature mean value of the monitoring object, and store the 128D feature mean value locally. When the system is working, recognize the face in the video stream, extract the feature points in the face and store the local face image information to match the Euclidean distance to determine whether it is the same face; if so, return the corresponding person identity information, if not, it displays unknown. When the threshold set for face recognition is 0.4 and the Euclidean metric matching degree is less than or equal to 0.4, return the corresponding character identity information, and face recognition is successful.

In Step 6, consider the food taboos of users, such as the following: seafood-allergic people do not eat seafood; Hui people do not eat pork; vegetarians do not eat meat, eggs and milk; and pregnant women are not allowed to eat cold foods. As a result, build a taboo food database G (see Table 3).

## 5. Experimental Environment

**Figure 2:**
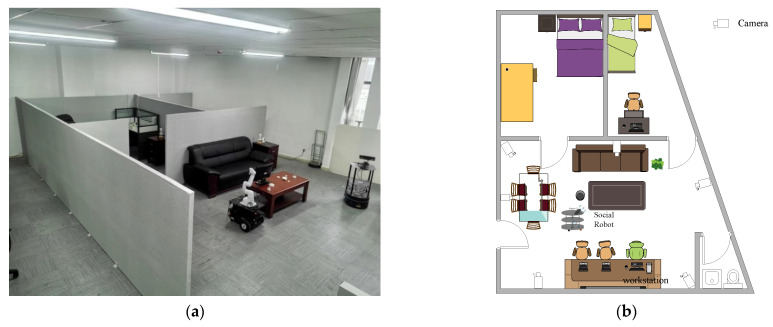
Smart home experimental environment. (**a**) The built experimental environment. (**b**) Floor plan of experimental environment.

The smart home experimental environment built in this paper is shown in Figure 2; in this setting, multiple cameras and a social robot with a depth camera were deployed to monitor the user’s dietary behavior, and the frame data captured from multiple camera video streams at the same moment were transmitted to the workstation in real-time through wireless communication, while the training and analysis of the data were performed by a Dell Tower 5810 workstation (Intel i7-6770HQ; CPU, 2600 MHz; 32G memory. NVIDIA Quadro GV100 GPU; 32G memory) [42,43]. The hardware of the social robot included an Intel NUC mini host, EAI DashgoB1 mobile chassis, IPad display screen and Microsoft Kinect V2 depth camera, and the communication control between hardware modules was implemented using the ROS (robot operation system) framework [44]. At the software level, the social robot’s platform host and workstations were installed with the Ubuntu 16.04 LTS operating system, TensorFlow deep learning framework, YOLO and machine vision toolkit Opencv3.3.0.

**Figure 3:**
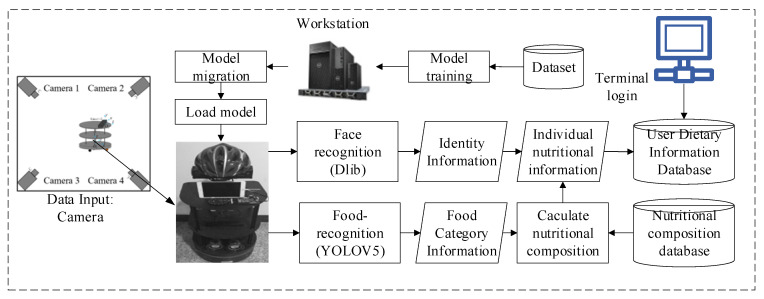
Overall workflow of the autonomous perception system for dietary nutritional information in smart homes.

Figure 3 shows the workflow chart of the autonomous perception system for dietary nutritional information in a smart home environment. First of all, the Dell Tower 5810 workstation uses Algorithm 1 to train the food image dataset to obtain the food-recognition feature model *w*. Then, the obtained feature model *w* is transmitted to the social robot, which receives the model and loads it, and the multiple cameras deployed to the smart home environment and the social robot with depth cameras apply DNPM to start food-recognition detection while importing the face data feature database for face recognition. Finally, the food category information is mapped to the nutritional composition database according to the detected results, the nutritional composition is calculated (see Section 4), and the nutritional composition information of the user is obtained and stored in the user dietary information database. Users can query their dietary information through the terminal.

## 6. Performance Experiment and Analysis of Food-Recognition Algorithm

### 6.1. Dataset

The 23 most common kinds of food were selected from the ChinesFoodNet [27] dataset as the training set and test set, including cereal, potato, vegetable, meat, egg, milk and seafood. Considering that the food type in the actual scenario should also include milk and fruit, milk and 10 kinds of fruits were added to expand the dataset; in total, 34 kinds of food images were formed, and the dataset CFNet-34 was formed. We took 80% of the CFNet-34 dataset as the training dataset and 20% as the test dataset for training and testing, respectively. Dataset acquisition address: https://pan.baidu.com/s/1laUwRuhyEEOmWq8asi0uoA, (accessed on 19 June 2022) Extraction code: 71l4.

### 6.2. Performance Indicators

Four indicators of precision rate P (see Equation (2)), recall rate R (see Equation (3)), *mAP@0.5* and *mAP@0.5:0.95* were used to evaluate the food-recognition model.

(2) $$P=Precision=\frac{TP}{TP+FP}.$$

(3) $$R=Recall=\frac{TP}{TP+FP}.$$

where *TP_i_* represents the number of foods of category *i* that are correctly predicted, *N* represents the total number of categories of foods, *FP_i_* represents the number of other foods that are incorrectly predicted as foods of category *i*, and *FN_i_* represents the number of foods of category *i* that are incorrectly predicted as other foods.

*mAP@0.5* represents the *mAP* when the IoU threshold is 0.5, reflecting the recognition ability of the model. *mAP@0.5:0.95* represents the average value of each *mAP* when the IoU threshold is from 0.5 to 0.95 and the step size is 0.05, which reflects the localization effect and boundary regression ability of the model. The values of these six evaluation indicators are all positively correlated with the detection effect. *AP* in *mAP* is the area under the *PR* curve, and its calculation method is shown in Equation (4).

(4) $$AP=\int_{0}^{1}Precision\left(Recall\right)dRecall.$$

### 6.3. Experimental Results and Analysis

The hyperparameters of the experiment were set as follows: iteration times, 600; batch size, 32; learning rate, 0.001; size of all input images, 640; confidence threshold, 0.01; IoU threshold, 0.06; and the test set was tested.

Table 4 shows the evaluation results of food recognition obtained by testing the YOLOv5 model on the test set. Obviously, the more obvious the image features, the easier they were to identify. For example, the recognition accuracy of fruits was higher, and the recognition accuracy of strawberries with the most obvious features reached 100%. The inter-class similarity among three kinds of dishes, i.e., braised pork, barbecued pork and cola chicken wings, is too large, which can easily lead to recognition errors. Therefore, the recognition accuracy was low, and the recognition accuracy of cola chicken wings was the lowest, at 69.5%. The average accuracy of the model test was 89.7%, the average recall rate was 91.4%, and the average *mAP@0.5* and *mAP@0.5:0.95* were 94.8% and 87.1%, respectively.

Table 5 shows the experimental results of different image recognition algorithms on the test set. It can be seen from Table 5 that Algorithm 1 performs well on the whole, and the Top-1 and Top-5 accuracy rates of the test set are higher than other algorithms, and a more robust feature model can be obtained, thereby improving the recognition accuracy of the algorithm. It shows that Algorithm 1 has higher recognition accuracy and robustness in food recognition.

## 7. Application System Performance Testing and Analysis

### 7.1. Experiment Solution

See Table 6 for indications to set test scenarios considering the possible number of family members and the number of foods.

In order to test the food recognition and nutritional composition perception performance of the system, seven types of test sets were designed from the aspects of test object change, food change, etc.

Test set a: There was only one kind of food in the sample image, and the sample image was divided into six categories, including cereal, potato, vegetable, meat, egg, milk, seafood and fruit. Each category had 10 images, for a total of 60, and did not intersect with the training set.

Test set b: There were 60 images with two kinds of food in the sample image.

Test set c: There were 60 images with three kinds of food in the sample image.

Test set d: There were 60 images with four kinds of food in the sample image.

Test set e: There were 60 images with six kinds of food in the sample image.

Test set f: There were 60 images with eight kinds of food in the sample image.

Test set g: There were 60 images with nine kinds of food in the sample image.

The working parameters of the camera are not easy to calculate, so the test set used in the test is usually prepared in advance, and the data is sent to the system by simulating the working mechanism of the camera.

### 7.2. Test Results and Analysis

**Figure 4:**
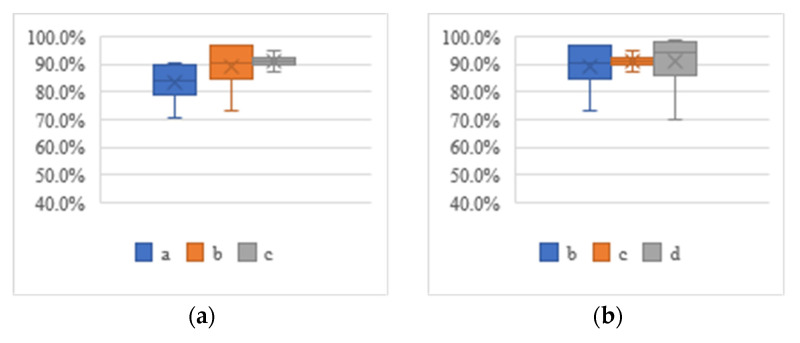

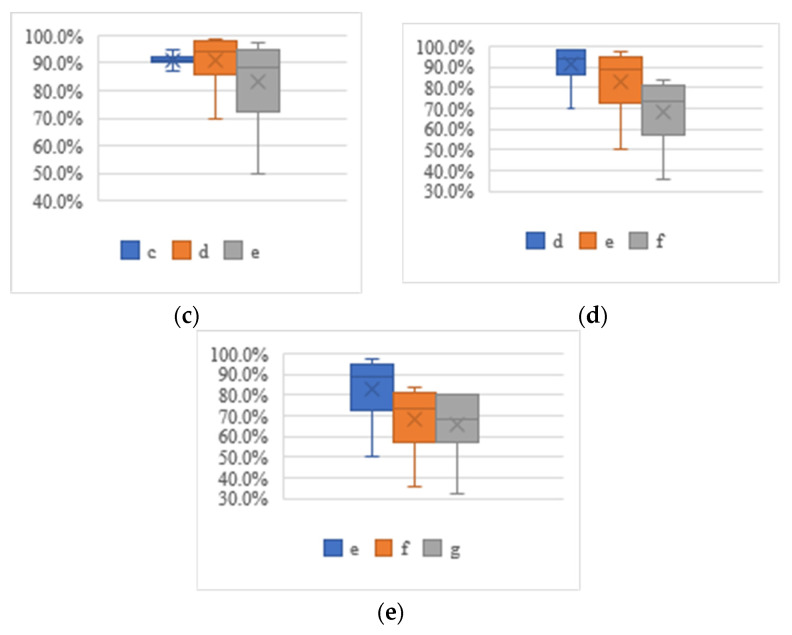
Box plots of nutritional composition perception accuracy for test sets of different scenarios. (**a**) C_1_ scenario test set. (**b**) C_2_ scenario test set. (**c**) C_3_ scenario test set. (**d**) C_4_ scenario test set. (**e**) C_5_ scenario test set.

**Figure 5:**
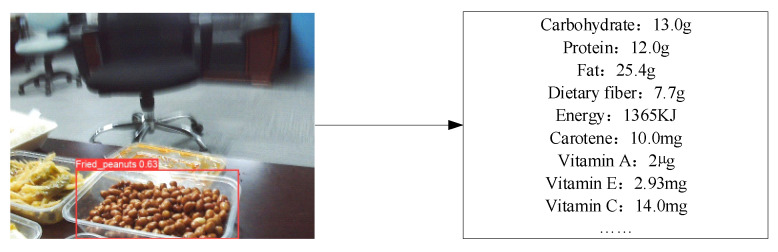
System diet evaluation effect chart.

When the proposed algorithm was deployed on the social robot platform, the hyperparameters were set as follows: number of iterations, 600; batch size, 32; learning rate, 0.001. The five scenarios and seven types of test sets designed in Section 7.1 were tested. The response time and speed of the system for different test sets and the perceived accuracy of nutritional composition are shown in Table 7, Table 8 and Table 9. The box diagram of nutritional composition perception accuracy is shown in Figure 4. The effect chart of the systematic diet assessment is shown in Figure 5.

After food recognition and face recognition, Algorithm 2 can be used to quickly correlate food nutritional information, so the accuracy of food recognition is the perception accuracy of the nutritional composition.

According to Table 7, the average response time of the system was 4.6 ms, and the average response times of test set a ~ test set g were 3.8 ms, 4.1 ms, 4.5 ms, 4.6 ms, 4.9 ms, 5.1 ms and 5.5 ms, respectively. The average response time of the system for different test sets increased with the increase in personnel and food, indicating that the detection and recognition of the system was more time-consuming in the scenario with more food and personnel; however, the response speed is in the millisecond range, which meets the real-time working requirements of the system.

According to Table 8, the average recognition speed of the system was 21.8 fps, and the average recognition speeds of test set a ~ test set g were 26.3 fps, 24.4 fps, 22.0 fps, 21.6 fps, 20.3 fps, 19.7 fps and 18.2 fps, respectively. According to Table 9, the total average nutritional composition perception accuracy of the system was 90.1%, and the average nutritional composition perception accuracy values of test set a ~ test set g were 89.7%, 92.5%, 93.3%, 97.2%, 96.5%, 80.9% and 80.3%, respectively. In the scenario where 3 or 4 people eat four foods and six foods, the nutritional composition perception of the system was the most accurate, while in the case of more food and personnel, the performance of the system was affected to a certain extent, but on the whole, the nutritional composition perception accuracy of the system was good.

According to Figure 4, the median scale of Figure 4a–c is higher than 80.0%, indicating that the system showed good recognition performance for the data of the C_1_, C_2_ and C_3_ scenarios, while Figure 4d,e indicates that the lowest-value scale line is close to 30.0%, indicating that the system showed poor recognition performance for the data of the C_4_ and C_5_ scenarios. In general, the nutritional composition perception accuracy of the system was 90.1%, but in the case of complex personnel and food, the recognition and perception performance of the system was low; therefore, the recognition and perception robustness of the system needs to be further improved.

To sum up, the test set proved that the response time of food recognition and face recognition of the system for different test sets was less than 6 ms, and the speed was higher than 18 fps. The overall nutritional composition perception accuracy of the system was 90.1%, indicating that the feature model output of this algorithm has a certain generalization ability, the algorithm has a strong feature-learning ability, and the system has good robustness.

## 8. Discussion

Though our proposed algorithm performs well on self-built datasets, there is room for improvement compared with some state-of-the-art algorithms.

Model complexity has always been a major factor affecting the performance of deep learning models. Due to hardware limitations, we need to make a trade-off between processing time and system accuracy. In the experiments, we use YOLOv5 for food recognition. YOLOv5 is the most advanced target detection method at present, but the training process is time-consuming and the accuracy of target detection needs to be improved. In the future, we may try to improve the YOLOv5 model structure in terms of reducing training time and increasing recognition accuracy, such as further combining the feature fusion of each module with multi-scale detection [45] and introducing attention mechanism modules in different positions of the model [46].

The second challenge is to generate a good dataset that we can use to capture food images from our daily diet. As the problem that we encountered in our evaluation, though we have the popular image dataset ChineseFoodNet dataset, some images in the dataset are inaccurately classified. Otherwise, some food items have high intra-class variance or low inter-class variance. Items in the same category with high intra-class variance might look different, and two different types of food with low inter-class variance have similar appearances. Both high intra-class variance and low inter-class variance issues can significantly affect the accuracy of the detection model. To solve this problem, we need to search more datasets to augment the CFNet-34 dataset. In the future, we will continue to label our CFNet-34 dataset to extend this dataset to a wider range of food categories. Combinations with other datasets to create a more diverse food dataset are desirable.

## 9. Conclusions

In order to reduce the risk of disease caused by the user’s obesity and being overweight and to regulate the user’s dietary intake from the perspective of dietary behavior, it is necessary to develop a social robot with functions of dietary behavior monitoring and dietary quality assessment. Focusing on the needs of users’ dietary behavior monitoring and diet quality assessment in the smart home environment, this paper proposes a dietary nutritional information autonomous perception method based on machine vision in smart homes. The method applies deep learning, image processing, database storage and management and other technologies to acquire and store the user’s dietary information. Firstly, we proposed a food-recognition algorithm based on YOLOv5 to recognize the food on the table. Then, in order to quantitatively analyze the user’s dietary information, we calibrated the weight of the food ingredients and designed a method for the calculation of the nutritional composition of the foods. Based on this, we proposed a dietary nutritional information autonomous perception method based on machine vision (DNPM) to calculate the user’s nutritional composition intake. The acquired user dietary information is stored in the autonomous perception system of dietary nutritional information for the user to query. Finally, the proposed method was deployed and tested in the smart home environment. The test results show that the system response time of the proposed method was less than 6 ms, and the nutritional composition perception accuracy rate was 90.1%, showing good real-time performance, robustness and nutritional composition perception performance. However, this study needs to be further strengthened. Firstly, social robots lack the ability to dynamically and autonomously add food and people. In addition, the user and the social robot do not establish a stable human–machine relationship only through face recognition. In future research, we want to focus on the functional design of social robots to autonomously add food and people and to build a stable relationship between humans and machines. In addition, we will continue to work to improve the accuracy of system recognition and reduce system processing time.

## Figures and Tables

**Table 1:** A total of 32 basic ingredients and their calibrated quantities.

Vegetables, Potatoes, Fruits					Meat, Eggs, Dairy	Seafood	Whole Grains
Sweet potatoes, 200 g	Cabbage, 250 g	Potatoes, 200 g	Tomato, 250 g	Cauliflower, 300 g	Chicken, 500 g	Fish, 500 g	Tofu, 200 g
Green peppers, 200 g	Vermicelli, 150 g	Water spinach, 250 g	Eggplant, 300 g	Oranges, 250 g	Pork, 300 g	Shrimp, 300 g	Rice, 200 g
Caster sugar, 50 g	Cantaloupe, 250 g	Peaches, 250 g	Pears, 250 g	Cherries, 250 g	Eggs, 150 g	-	Peanuts, 100 g
Kiwi, 250 g	Mango, 250 g	Strawberries, 250 g	Banana, 250 g	Apple, 250 g	Cream, 100 g	-	Corn, 200 g
-	-	-	-	-	Milk, 100 g	-	Wheat flour, 150 g

**Table 2:** Nutritional composition of main ingredients of green pepper shredded pork.

Name	Weight (g)	Carbohydrates (g)	Protein (g)	Fat (g)	Dietary Fiber (g)	Cholesterol (mcg)	Energy (kJ)
Green pepper	200	11.6	2.8	0.6	4.2	0	266
Pork	300	7.2	39.6	111.0	0	240	4902
Total	500	18.8	42.4	111.6	4.2	240	5168
Carotene (mg)	Vitamin A (mcg)	Vitamin E (mg)	Vitamin C (mg)	Vitamin B1 (mg)	Vitamin B2 (mg)	Vitamin B3 (mg)	Vitamin B6 (mg)
680.0	114	1.76	124.0	0.06	0.08	1.00	4.60
0	54.0	0.60	3.7	0.66	0.48	10.50	1.35
680.0	168.0	2.36	127.7	0.72	0.56	11.50	5.95
Vitamin B9 (mcg)	Vitamin B12 (mcg)	Choline (mg)	Biotin (mcg)	Calcium (mg)	Iron (mg)	Sodium (mg)	Magnesium (mg)
87.60	0	0	0	30	1.4	4.4	30
2.67	1.08	0	0	18	4.8	178.2	48
90.27	1.08	0	0	48	6.2	182.6	78
Phosphorus (mg)	Manganese (mg)	Copper (mg)	Potassium (mg)	Selenium (mcg)	Zinc (mg)	Fatty Acids (g)	
66	0.28	0.22	418	1.20	0.44	0	
486	0.09	0.18	612	36.00	6.18	0	
552	0.37	0.40	1030	37.20	6.62	0	

**Table 3:** Taboo foods.

Taboo Food	Group	Related Food	Related Dishes
Seafood	Seafood Allergy	Fish, Shrimp, Crab and Shellfish	Braised Prawns, Steamed Fish
Meat	Vegetarian	Pork, Beef, Mutton, Chicken, Duck, Fish, Shrimp, Crab Shells, Eggs, Milk	Green Pepper Shredded Pork, Barbecued Pork, Braised Pork, Corn Rib Soup, Tomato Scrambled Eggs, Steamed Egg Drop, Spicy Chicken, Braised Prawns, Steamed Fish
Pork	Hui People	Pork	Green Pepper Shredded Pork, Char Siew, Braised Pork, Corn Pork Rib Soup
Cold Food	Pregnant	Lotus Root, Kelp, Bean Sprouts, Water Spinach, Vermicelli, Duck Eggs, Duck Blood, Duck Meat, Crab, Snail, Soft-Shelled Turtle, Eel, Banana, Cantaloupe, Persimmon, Watermelon and Other Fruits	Garlic Water Spinach, Fried Dough Sticks, Hot and Sour Powder

**Table 4:** Recognition evaluation of food test set.

Food Category	*Precision*	*Recall*	*mAP@0.5*	*mAP@0.5:0.95*
Candied Sweet Potatoes	0.842	0.900	0.941	0.826
Vinegar Cabbage	0.835	0.838	0.889	0.816
Char Siew	0.764	0.688	0.811	0.695
Fried Potato Slices	0.923	0.988	0.990	0.91
Scrambled Eggs with Tomatoes	0.793	0.988	0.965	0.911
Dry Pot Cauliflower	0.967	0.724	0.924	0.832
Braised Pork	0.726	0.925	0.918	0.800
Cola Chicken Wings	0.695	0.799	0.794	0.676
Spicy Chicken	0.882	0.937	0.958	0.853
Rice	0.971	0.844	0.961	0.824
Mapo Tofu	0.840	0.975	0.986	0.940
Green Pepper Shredded Pork	0.794	0.913	0.926	0867
Cookies	0.917	0.850	0.933	0.786
Hot And Sour Powder	0.847	0.937	0.970	0.892
Garlic Water Spinach	0.900	0.895	0.958	0.909
Garlic Roasted Eggplant	0.857	0.759	0.894	0.789
Small Steamed Bun	0.891	0.814	0.896	0.734
Fried Shrimps	0.943	0.962	0.977	0.916
Corn Rib Soup	0.948	0.975	0.988	0.922
Fritters	0.824	0.886	0.881	0.751
Fried Peanuts	0.960	0.938	0.967	0.907
Steamed Egg Drop	0.735	0.963	0.950	0.860
Steamed Fish	0.927	0.945	0.974	0.783
Milk	0.884	0.728	0.833	0.603
Cantaloupe	0.988	0.988	0.993	0.993
Peach	0.904	0.988	0.990	0.966
Pear	0.992	0.988	0.992	0.957
Cherry	0.991	0.988	0.993	0.988
Orange	0.991	0.988	0.993	0.993
Kiwi	0.991	1	0.995	0.990
Mango	0.989	1	0.995	0.995
Strawberry	1	1	0.995	0.982
Banana	0.993	1	0.995	0.944
Apple	0.98	0.975	0.994	0.993
Mean	0.897	0.914	0.948	0.871

**Table 5:** Top-1 and Top-5 accuracy rates of different image recognition algorithms on the test set.

Algorithm	Test Set	
	Top-1 Accuracy (%)	Top-5 Accuracy (%)
Squeezenet	62.36	90.26
VGG16	78.45	95.67
ResNet	77.24	95.19
DenseNet	78.12	95.53
This paper	80.25	96.15

**Table 6:** Test scenario settings.

No.	Scenario Information
C_1_	1 person eats 1–3 foods
C_2_	2 people eat 2–4 foods
C_3_	3 people eat 3, 4, 6 foods
C_4_	4 people eat 4, 6, 8 foods
C_5_	5 people eat 6, 8, 9 foods

**Table 7:** Statistical results of the response time of the system for different test sets.

Test Set	System Response Time in Different Scenarios (ms)					Mean
	C_1_	C_2_	C_3_	C_4_	C_5_	
a	3.8	-	-	-	-	3.8
b	4.0	4.2	-	-	-	4.1
c	4.5	4.5	4.6	-	-	4.5
d	-	4.5	4.7	4.7	-	4.6
e	-	-	4.7	4.9	5.2	4.9
f	-	-	-	4.9	5.3	5.1
g	-	-	-	-	5.5	5.5

**Table 8:** Statistical results of recognition speed of the system for different test sets.

Test Set	Recognition Speed in Different Scenarios (fps)					Mean
	C_1_	C_2_	C_3_	C_4_	C_5_	
a	26.3	-	-	-	-	26.3
b	25.0	23.8	-	-	-	24.4
c	22.2	22.2	21.7	-	-	22.0
d	-	22.2	21.3	21.3	-	21.6
e	-	-	21.3	20.4	19.2	20.3
f	-	-	-	20.4	18.9	19.7
g	-	-	-	-	18.2	18.2

**Table 9:** Statistical results of nutritional composition perception accuracy for different test sets.

Test Set	Nutritional Composition Perception Accuracy in Different Scenarios (%)					Mean
	C_1_	C_2_	C_3_	C_4_	C_5_	
a	89.7	-	-	-	-	89.7
b	96.8	88.2	-	-	-	92.5
c	91.4	94.7	93.8	-	-	93.3
d	-	98.5	94.3	98.9	-	97.2
e	-	-	97.2	94.2	98.1	96.5
f	-	-	-	83.3	78.4	80.9
g	-	-	-	-	80.3	80.3

## Data Availability

https://pan.baidu.com/s/1laUwRuhyEEOmWq8asi0uoA, (accessed on 19 June 2022) Extraction code: 71l4.
